# Lacrimal gland mucoepidermoid carcinoma with contralateral eye and systemic metastasis: A rare case report and review of the literature

**DOI:** 10.3389/fonc.2023.1131587

**Published:** 2023-03-09

**Authors:** Yi Wu, Weimin He

**Affiliations:** Department of Ophthalmology, West China Hospital, Sichuan University, Chengdu, Sichuan, China

**Keywords:** mucoepidermoid carcinoma, lacrimal gland, contralateral eye, recurrence, multifocal metastasis, diagnosis, therapy, case report

## Abstract

Lacrimal gland mucoepidermoid carcinoma is very rare. It has a high risk of recurrence and metastasis, however, it rarely metastasizes to the contralateral eye in clinical practice. Here, we present a case of a 52-year-old man with lacrimal gland mucoepidermoid carcinoma who developed multiple recurrences and metastases of another eye and other sites throughout the body after receiving surgical intervention and regular radiotherapy, which will be of ophthalmic interest and unique. Clinical features, imaging findings, histopathology, treatments, and outcomes of this very rare case are provided. A literature review of previously published cases of this disease is performed, with an emphasis on the latest diagnosis and treatment. The prognosis of tumor recurrence and metastasis is poorer, surgery with a negative margin in conjunction with adjuvant therapies is crucial for preventing local recurrence and distant metastasis and enhancing the survival rate.

## 1 Introduction

Mucoepidermoid carcinoma (MEC), first described by Stewart in 1945 (1), is the most common primary epithelial tumor of the salivary glands in both adults and children but extremely rare in the lacrimal gland (LG) (2). Women in their middle and older years are more likely to develop LG MECs, which lack any differentiating clinical or radiological features. MEC is prone to local recurrence and distant metastasis that most often occur to the parotid gland, lung, brain, and mediastinum (3). To the best of our knowledge, few cases of metastasis to the contralateral eye have been reported. Thus, a correct diagnosis and systemic management pose a challenge for an ophthalmologist. It possesses distinctive histological characteristics and constitutes the benchmark for diagnosis. In recent years, some immunological and molecular markers have been discovered to aid in diagnosis. Optimal favorable therapeutic protocols for LG MECs are still being developed, and the treatments currently in use are based on those for MECs of the salivary gland. We now present the first case of LG MEC metastasis to the contralateral eye, including its clinical features, imaging findings, histopathology, treatments, and outcomes, and briefly review the literature.

## 2 Case presentation

**Figure 1:**
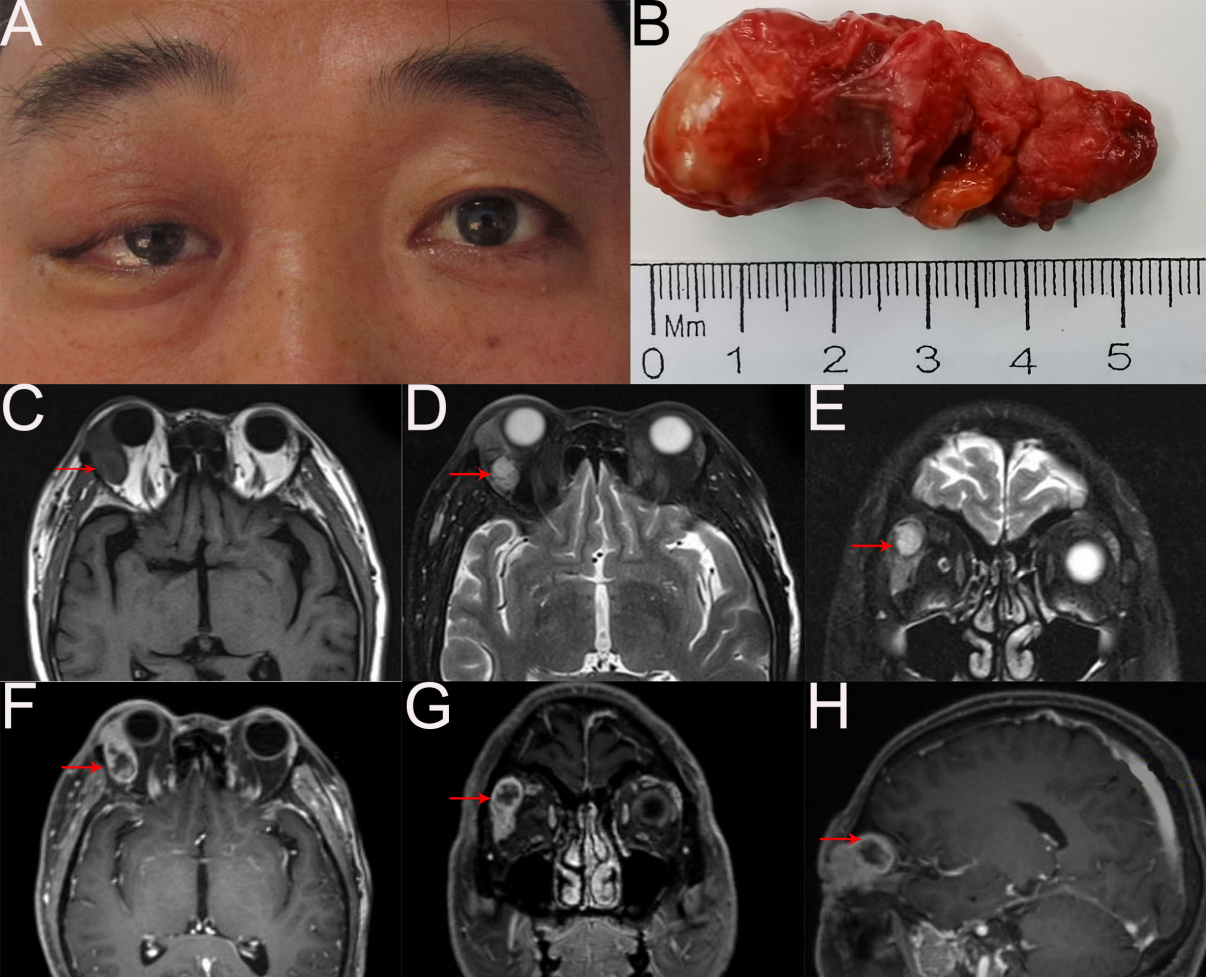
**(A)** External image of the patient. **(B)** The mass was dissected under the table and was approximately 5.5cm x 2.3cm x 1cm in size. **(C–H)** Orbital MRI scan revealed right lacrimal gland was enlarged obviously and an analogous round mixed-signal (2.3cm×2.0cm×1.5cm) shadow in the right lateral posterior superior orbit with heterogeneous enhancement. T1-weighted imaging without contract enhancement and fat suppression **(C)**. T2-weighted imaging with contract enhancement and fat suppression **(D)** and **(E)**. T1-weighted imaging with contract enhancement and fat suppression **(F-H)**.

A 52-year-old man presented with a 2-month history of a mass in the right upper eyelid (**Figure 1A**). On ophthalmology examination, a firm, well-circumscribed, nonmobile soft mass in the right orbit was palpated, the right upper eyelid was swollen and dropped to cover the 4 mm cornea, and the ocular movement was restricted in the superior direction. The best corrected visual acuity was 20/20 in both eyes. The remainders of ophthalmic examination results were normal. There are no unusual medical conditions or cancers in the family. Contrast-enhanced magnetic resonance imaging (MRI) of the orbit revealed that the right lacrimal gland was significantly enlarged, and an analogous round mass in the right lateral posterior superior orbit with heterogeneous enhancement; no obvious abnormalities were found in the left eye (**Figures 1C–H**). General examination showed no metastasis.

**Figure 2:**
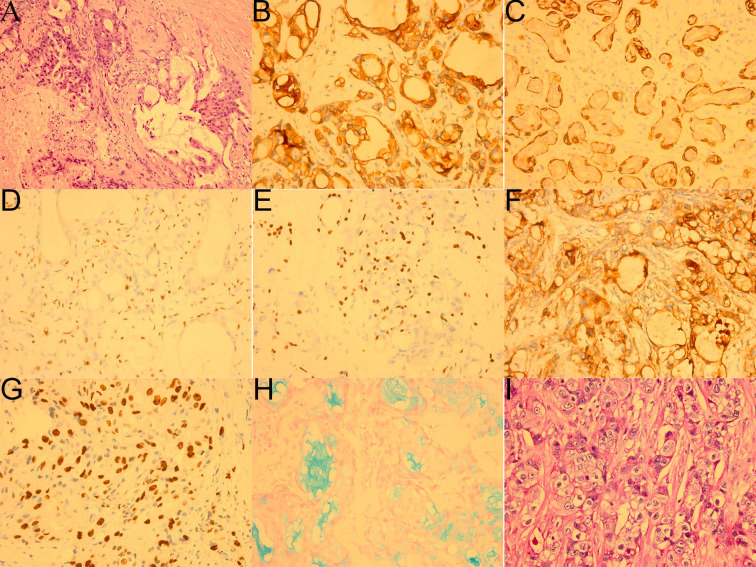
Pathological images of the patient`s first operation (. **(A)** HE showed the tumor was mainly composed of squamous epidermoid cells, intermediate cells and mucinous cells with tumor necrosis (bottom left, ×200). **(B)** Immunohistochemical staining CK7 (+,×400). **(C)** Immunohistochemical staining CK5/6 (+,×400). **(D)** Immunohistochemical staining p40 focal (+,×400). **(E)** Immunohistochemical staining p63 focal (+,×400). **(F)** Immunohistochemical staining CEA (+,×400). **(G)** Immunohistochemical staining Ki-67 (+, 30%,×400). **(H)** AB focal (+,×400). **(I)** PAS focal (+,×400).

After doing the required preoperative exams, a thorough review and signed informed consent outlining the operation’s risks was acquired. We first entered the orbit from the superolateral orbital margin, and opened the orbital septum to fully exposed the mass, then removed the mass and periorbita to verify the negative surgical margin. Intraoperatively, the mass was found to be tightly adhered to the levator palpebrae superioris muscle, superior fornix conjunctiva and lateral tarsal ligament. It was situated in the right lacrimal fossa and extended posteriorly to the orbital apex. While the retrobulbar portion was dark red and pale yellow with an ambiguous boundary, the lacrimal fossa portion was gray and oval with a clear boundary (**Figure 1B**). Subsequently, the hematoxylin-eosin (HE) revealed the tumor was primarily made up of epidermoid cells, intermediate cells and mucinous cells (**Figure 2A**). Immunohistochemical staining revealed that CK7、CK20、CK5/6、p63、p40 and CEA were positive whereas CD117、CD56、S-100 were negative, the Ki67 proliferation index was around 30%; in addition, the alcian blue staining (AB) and periodic acid-schiff stain (PAS) were positive (**Figures 2B–I**). Even though FISH did not detect a *MAML2* gene translocation, the aforementioned features were enough to validate the diagnosis of high-grade mucoepidermoid carcinoma. Two months following surgery, the patient received modulated intensity radiation therapy (IMRT) at a total dosage of 5992cGy with daily doses of 214cGy (28 fractions in a month).

**Figure 3:**
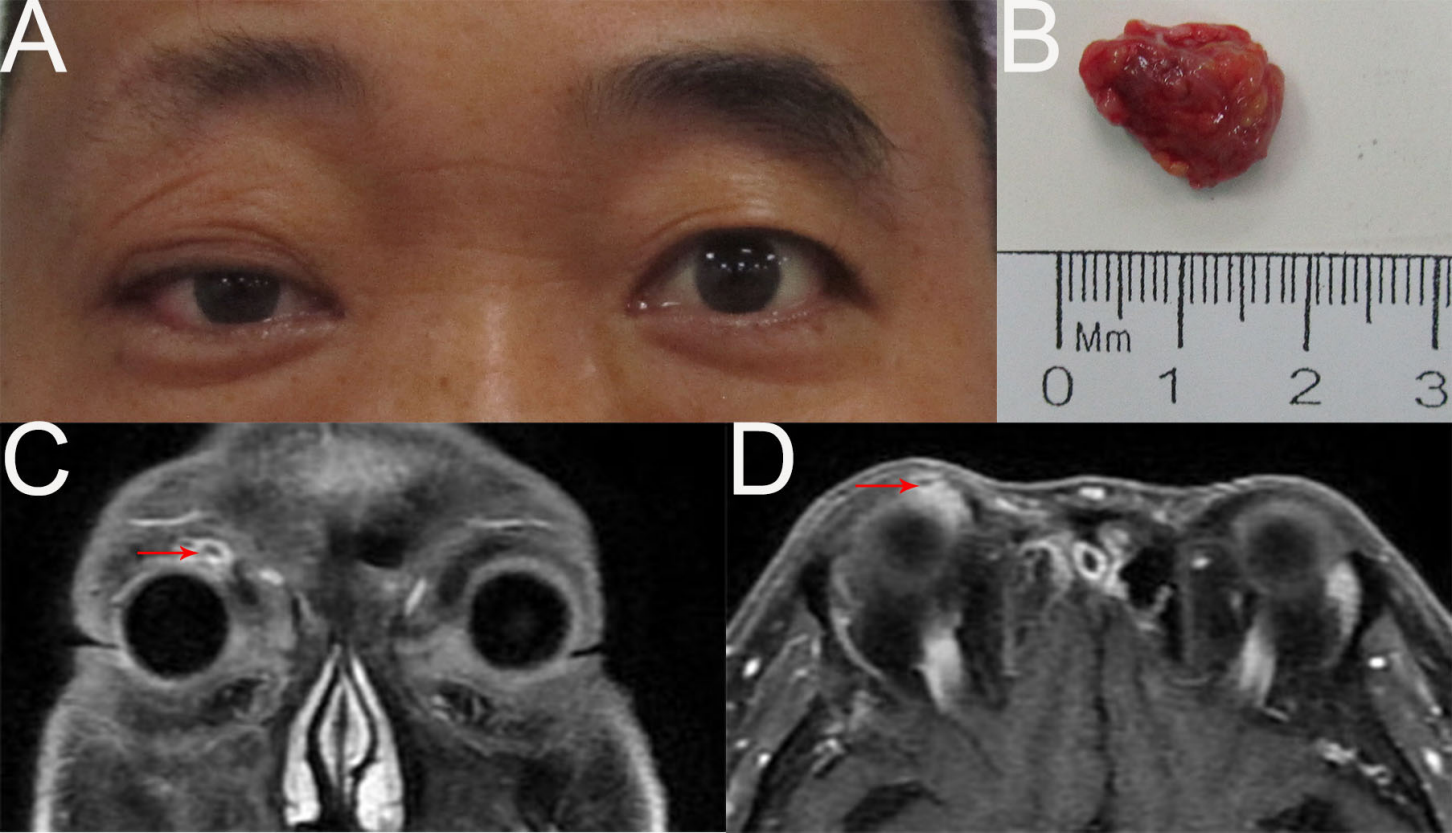
**(A)** External image of the first recurrence. **(B)** The mass was dissected under the table and was approximately 1.7cmx1.2cmx0.8cm in size. **(C, D)** Contract-enhanced MRI with STIR sequence of the orbit showed the absence of the right lacrimal gland and an oval abnormal signal (1.0cm×0.9cm) in the right superomedial orbit with peripheral rim enhancement.

Eight months later, the patient returned with a right orbital mass (**Figure 3A**). A poor-defined, nonmobile soft mass was found in the right superomedial orbit. An orbital MRI scan revealed a mass in the right superomedial orbit with peripheral rim enhancement, and no evident abnormalities in the left eye (**Figures 3C, D**). A submandibular gland nodule seen by neck ultrasonography raised the possibility of malignant spread. For reasons of prognosis, disfigurement, and vision loss, the patient chose tumor resection instead of orbital exenteration (**Figure 3B**). The pathological diagnosis was recurrent MEC. The patient was advised to receive chemoradiotherapy subsequently, but he refused because the last radiotherapy caused vision loss in his right eye. Two months after surgery, an additional hospital’s PETCT scan revealed a tumor recurrence in the right orbit and multiple metastases in the right submandibular gland, parotid gland, neck, bilateral frontal sinuses, ethmoid sinuses, maxillary sinuses, mandible, mediastinum, bilateral lung, intracranial, and systemic lymph nodes. Due to extensive metastases, the patient stopped receiving treatment.

**Figure 4:**
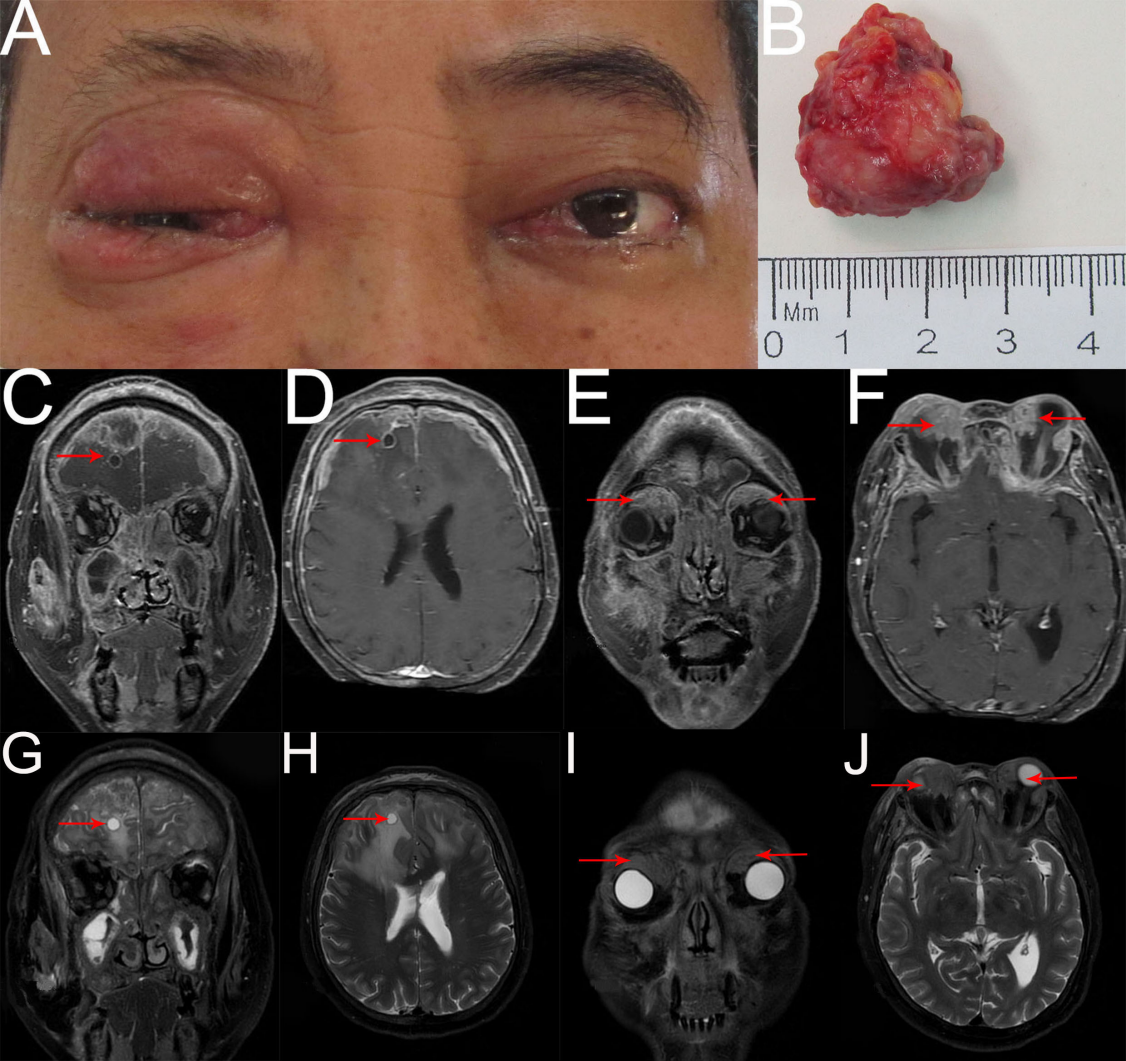
**(A)** External image of the second recurrence. **(B)** The mass was dissected under the table and was approximately 2.8cmx2.5cmx1.3cm in size. **(C–J)** Contract-enhanced MRI with STIR sequence of the orbit showed two irregular mixed-signal shadows on the right superomedial orbit and the left superior orbit with mild enhancement (**E, F, I, J** red arrow). Bilateral frontal meninges were thickened and enhanced obviously, and part of them had liquefaction necrosis. An analogous round mass with peripheral rim enhancement was seen in the right frontal lobe, and significant edema with unclear boundaries was observed in bilateral frontal lobes and corpus callosum (**C, D, G, H** red arrow). Additionally, numerous irregular abnormal signals with heterogeneous enhancement were observed in bilateral ethmoid sinuses, maxillary sinuses, sphenoid sinuses, frontal sinuses, right infratemporal fossa, face, zygomatic area, and parotid region, involving some bone.

**Figure 5:**
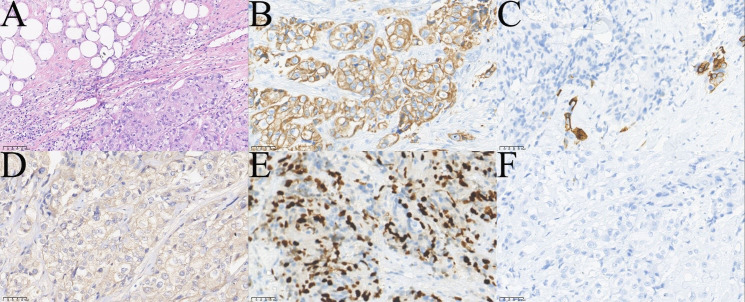
Pathological images of the patient`s fourth operation. **(A)** HE showed infiltrating tumor cells mainly consisted of squamous epithelial and clear cells (top left of A,×200). **(B)** Immunohistochemical staining CK7 (+,×400). **(C)** Immunohistochemical staining showed a few cells CK5/6 (+,×400). **(D)** Immunohistochemical staining Ber-EP4 (+,×400). **(E)** Immunohistochemical staining Ki-67 (+, 40%,×400). **(F)** Immunohistochemical staining p63(-,×400).

Two months later, the patient returned again with gait instability caused by cerebral metastasis, an MRI scan revealed an analogous round mass with peripheral rim enhancement in the right frontal lobe, diffuse thick pachymeningeal enhancement along bilateral frontal lobes, and widespread edema in both frontal lobes and corpus callosum (**Figures 4C, D, G, H**). It also revealed two masses, one in the right superomedial orbit and the other in the left superior orbit, which indicated the recurrence and metastasis of an orbital tumor (**Figures 4E, F, I, J**). He then had brain gamma knife therapy with a 15Gy dosage and a 45% isodose cure. Two months later, due to the intense pain in the left eye, the patient returned for treatment to our department (**Figure 4A**) and had the tumor in the left orbit resected. Intraoperatively, a grayish-white neoplasm was found in the left superior orbit with the orbital roof and levator palpebrae superioris muscle involved (**Figure 4B**). The pathological diagnosis was high-grade metastatic MEC (**Figure 5**). After consultation with oncologists, the patient was advised to pursue additional therapy, but he declined due to the high risk and unfavorable effects of chemoradiotherapy. Eventually, the patient died of systemic metastasis 4 months after the last surgery, as reported by telephone follow-up.

## 3 Discussion

LG MECs are rare entities; it makes up 3.6% of all lacrimal malignant tumors (4). Fifty-two cases were mentioned globally from 1963 to 2022. Due to incomplete information in the literature, we reviewed forty-seven cases with available information (see **Supplementary Table 1**). There were 21 males and 26 females with age ranging from 12 to 79 years (median :48.4 years). In the group of 37 patients whose eye laterality was known, were 17 right eyes and 20 left eyes; there were no bilateral cases. The tumor’s cause is uncertain, possibly relating to ionizing radiation, gene translocation, malignant transformation of pleomorphic adenoma, HPV6 and HIV infection (2, 5–8). This case is peculiar in that the presentation is in a middle-aged man who has metastasis to multiple organs including the contralateral eye.

MEC, whether it is primary or metastatic, typically manifests as a slow-growing painless tumor mass, while pain from nerve invasion is possible in some case. The presentation of our patient with a unilateral orbital mass associated with eyelid swollen, globe displacement, ptosis, pain, and limitation of movement is in keeping with previous reports. Additionally, some patients have mentioned proptosis, diplopia, visual loss, tearing, redness, dryness, photophobia, choroidal folds (5–7, 9–11), and a few patients even have no symptoms (6, 12).

The diagnosis of MEC can only be confirmed histologically, immunohistochemical and molecular studies could aid in the diagnosis, but radiography is necessary for determining the location, size, and extent of the tumor, for detecting the cystic changes, bone invasion and metastasis, and for planning the extent of surgical intervention. MEC is usually cystic and consists of different proportions of epidermoid cells, intermediate cells and mucinous cells, sometimes including columnar cells and clear cells (13). According to Brandwein Schemes for Grading used for MEC of the salivary gland, the tumor is categorized as low, medium, and high grade, which could predict prognosis and conduct treatment (6). Low-grade tumors are well differentiated and contain more than 50% mucinous cells and squamous epithelial cells; high-grade tumors are poorly differentiated, mainly composed of squamous epithelial cells, intermediate cells and less than 10% mucinous cells; the histological features of medium-grade tumors are between low-grade and high-grade tumors (14, 15). Our patient’s expression of CK7, CK5/6, CK20, p63, p40, CEA, and positive results for AB and PAS is consistent with earlier studies. Other markers included CK8, EMA, vimentin, pan-keratin, HER2 and bcl-2. There were studies suggesting MEC was frequently associated with a translocation known as t(11;19)(q1421;p12-13), which led to the *CRTC1-MAML2* fusion gene (16). Although the fact that the *CRTC1-MAML2* fusion gene is not always found in LG MECs, as was seen in our case, its presence can be a decisive factor in the identification of LG MECs and assist with treatment planning (17).

Similar to the MEC of the salivary gland, the management of the lacrimal gland MEC depends on the histological grade. There were 19 low and intermediate-grade patients and 18 high-grade patients, and one patient with an oxyphilic variant in the group of 38 patients whose histological grade and outcome were known. Of 19 patients with low and intermediate-grade tumors, all generally showed a good prognosis, only one patient recurred as a high-grade tumor and died after 6 months, and one had metastases initially and died of brain metastasis after 2 years). Therefore, low-grade tumors rarely relapse and metastasize, and can be resected completely with or without radiotherapy. In contradistinction, the 18 patients with high-grade tumors generally had a diamal prognosis, only 8 patients were alive, including those who had residual cancer and inadequate follow-up (less than 1 year). Intracranial dissemination, lymph node and bone invasion, metastasis to parotid gland, lung, pelvis were responsible for the mortality of high-grade tumors. High-grade tumors generally carry a high risk of local recurrence and distant metastasis, Robinson et al. studied retrospectively 21 cases of MEC and suggested the recurrence rate of high-grade MEC is as high as 79% (18), and most often metastasizes to the parotid gland, lung, brain, mediastinum (3). As a result, these high-grade tumors require exenteration with radiotherapy and prompt resection of any involved orbital tissue. If there are systemic metastases, chemotherapy is necessary. Some authors argued for biopsy of lacrimal gland lesions first, however we believe that tumor excision is safer and more reasonable. Regardless of tumor grade, if the surgical margin is positive, recurrence and metastasis are more likely to happen and the prognosis is worse. Our patient experienced recurring recurrence and multiple metastases, which may be due to the high degree of tumor invasiveness, failure to obtain a negative surgical margin due to the complexity of the orbital structure, and refusion of adjuvant therapy. Postoperative radiation is recommended for high-risk patients with conditions such positive margins, high-grade tumors, lymph node metastases, nerve and vascular invasion (19, 20). The presence of the *CRTC1-MAML2* fusion gene is related to prognosis, Clauditz et al. reported that the frequency of *MAML2* rearrangement decreased significantly with the increase of tumor grade, which may predict a positive prognosis (21). Increasing patient age, tumor size(>4cm), male gender, extraparenchymal extension, node metastases, and distant metastases are additional negative prognostic variables (20).

According to the literature, the majority of occurrences of ocular tumor spread to the contralateral eye are malignant melanoma, with infrequent reports of retinoblastoma and lymphoma (22, 23). Bilateral primary tumors of different types are very uncommon, and our pathologic findings confirmed that both were MECs. Bilateral primary tumors of same types are also rare, and the diagnosis must be made in the absence of systemic metastases. Considering that the previous clinical scenarios are unlikely, it is presumed that the contralateral tumor may be a metastatic tumor from the affected eye or secondary spread of the systemic disease. Unfortunately, metastasis to the contralateral eye resulting from LG MECs generally occurs in patients with disseminated metastasis, so the treatment is palliative, even when presumed to be incomplete, may provide sufficient symptom relief for the rest of the patient’s life.

## 4 Conclusion

Although LG MECs are rare, they might be considered in the diagnosis of lacrimal gland tumors. The diagnosis should be made by combining histopathology, immunohistochemistry and *MAML2* rearrangement. Due to high-grade LG MEC being easy to relapse and metastasize, early diagnosis and timely, thorough surgical intervention combined with radiotherapy are of great importance to prevent local recurrence and distant metastasis. For metastatic MEC, palliative care is necessary and survival is poor. However, we also need further studies to explore immunotherapy and gene therapy to improve the survival rate.

## Data availability statement

The original contributions presented in the study are included in the article/**Supplementary Material**. Further inquiries can be directed to the corresponding author.

## Ethics statement

Written informed consent was obtained from the individual(s) for the publication of any potentially identifiable images or data included in this article.

## Author contributions

WH contributed to the implement of the treatment. YW and WH contributed to the collection and preparation of clinical data and graphic presentation. YW drafted the manuscript. WH supervised and reviewed the writing. All authors contributed to the article and approved the submitted version.
